# Modeling the supply chain sustainability imperatives in the fashion retail industry: Implications for sustainable development

**DOI:** 10.1371/journal.pone.0312671

**Published:** 2024-12-31

**Authors:** Md. Tariqul Islam Imran, Chitra Lekha Karmaker, Rubayet Karim, S. M. Misbauddin, A. B. M. Mainul Bari, Asif Raihan

**Affiliations:** 1 Institute of Nuclear Power Engineering, Bangladesh University of Engineering and Technology, Dhaka, Bangladesh; 2 Department of Industrial and Production Engineering, Bangladesh University of Engineering and Technology, Dhaka, Bangladesh; 3 Department of Industrial and Production Engineering, Jashore University of Science & Technology, Jashore, Bangladesh; 4 Institute of Forestry and Environmental Sciences, University of Chittagong, Chittagong, Bangladesh; National University of Sciences and Technology, PAKISTAN

## Abstract

The resilience of established business strategies has been tested in the wake of recent global supply chain upheavals triggered by events like the COVID-19 pandemic, Russia-Ukraine combat, Hamas-Israel war, and other geopolitical conflicts. Organizations are compelled to integrate sustainable practices into their supply chains to navigate the complexities of the post-COVID-19 era and mitigate the far-reaching consequences of such disruptions. However, exploring supply chain imperatives from sustainability dimensions still remains underexplored, presenting a significant research gap, particularly in the fashion retail sector. In response, this study aims to pioneer an innovative approach by amalgamating Pareto analysis, Bayes theorem, and the Best-Worst Method to evaluate sustainability imperatives comprehensively. Focusing on emerging economies like Bangladesh and its fashion retail industry, this methodology synthesizes insights from literature reviews, expert feedback, and Pareto analysis to curate a definitive set of influential imperatives. Finally, the Bayesian Best-Worst Method is applied to examine them. The results reveal the availability of government support schemes to promote sustainability, developing strategic supply chain interventions to ameliorate the impact of disruptive events, and digitalizing the supply chain as the most monumental imperatives under economic, social, and environmental perspectives, respectively. The study’s innovative methodology and its implications for sustainable supply chain management offer valuable insights for both academic research and practical application, presenting a strategic blueprint for the fashion retail industry to navigate and thrive in the post-COVID-19 era. This work can not only advance the theoretical understanding of supply chain sustainability but also provide actionable guidance for industry leaders in developing robust, resilient, and sustainable supply chain strategies.

## 1. Introduction

The global supply chain (SC) has encountered several phases of chronic disruptions, starting from the COVID-19 pandemic to the recent Russia-Ukraine war and war in Middle-eastern Asia, resulting in intermittence or halt in the production process and interrupted raw material supply [1, 2]. Wars have always brought adverse effects on the SC, fueled by production disruption, transportation outages, and supply disruptions [3]. Major fashion brands, e.g., Zara, Nike, and H&M, withdrew their operation from Russia in March 2022, which caused worry to fashion clothing manufacturers in Asia, including Bangladesh and Vietnam, where Asian nations will lose close to $1–2 billion or more in export profits [4]. The rising costs of crude oil and fuel have caused detrimental effects on the fashion SC as labor costs have increased due to inflationary pressures. The Russia-Ukraine war and the precedent of COVID-19 are leading to a global slowdown in the fashion industry [5]. The emergent energy crisis disrupted the fashion or clothing SC as 58% of executives think that the fashion market will weaken due to re-routing of trade, delay in delivery to European markets, use of sea instead of water, increase in freight expenses caused by sanctions on using seaports, and overall rising of cotton and cashmere prices by 30% to 45% [6].

From Bangladesh’s perspective, the war is feared to reduce the export-import business with Russia. In the fiscal year 2020–2021, Bangladesh exported goods worth USD 665.3 million to Russia, of which clothes were the most [7]. The disruption in the SC is expected to impact the Bangladeshi manufacturers due to the collapse in the SC resulting from heightened oil, gasoline, and ship fare costs. Because of the global value chain’s interconnected nature, disruption in one country has far-reaching effects on the overall international SC. More than 190,000 US and 109,000 European companies have tier-three Russian or Ukrainian suppliers [8]. Due to the sanctions, there have been widespread adverse effects on the SC operations of these suppliers. For example, the supply chains of electronics retailers have seen prices rise due to the reduced availability of rare earth metals from Russia and Ukraine. Supply chains of car retailers have also been impacted due to a reduction in the availability of palladium, which is used to produce vehicles’ catalytic converters [9]. The soaring cost and reduced energy availability have mostly affected energy-based supply chains [10]. These non-munificent, i.e., unfavorable environmental contingencies, require an enhanced emphasis by academicians and SC policymakers to ensure sustainability in the face of rampant SC disruptions [10]. In this respect, maintaining sustainability by accommodating a triple bottom line, e.g., social, economic, and environmental sustainability, can help firms maintain resilience and long-term viability [11]. Though several studies have been formulated in recent years considering the emergence of COVID-19, the imperatives leading to sustainability in the fashion retail SC have received very little attention. Primarily due to the new global paradigm shift resulting from the Russia-Ukraine war and recent SC adversities, it is time to focus on the imperatives contributing to sustainability, especially in the fashion retail industry.

In literature, sustainability issues have been primarily dealt with within the contexts of developed economies. However, there is limited understanding regarding how the fashion industry in an emerging economy can navigate SC disruptions and ensure sustainability in day-to-day operations. Considering this research and practice gap, the current study intends to explore the imperatives leading to sustainability in the fashion retail SC. Bangladesh is an attractive emerging economy where the readymade fashion retail industry is the prime export-earning sector. Bangladesh has secured a dominant position in the international fashion apparel chain due to the competitive advantages of low-cost labor. This sector’s contributions span various aspects, including economic growth, poverty alleviation, women empowerment, job opportunities, and many more. In the fiscal year 2022–2023, the clothing industry accounted for 84.58% of Bangladesh’s total export revenue, equivalent to approximately 46.99 billion USD [12].

Despite its significant role in the nation’s progress, Bangladesh’s fashion retail industry has faced numerous challenges since the early 1980s [13]. These challenges include the collapse of production facilities, political turmoil, and significant fire incidents. Notably, the tragic fire incident at Rana Plaza in 2014 highlighted the urgent need to ensure safe working conditions within the global retail sector [13, 14]. In response, two regulatory bodies, Accord and Alliance, were established to provide sustainable production practices [15]. While this sector has demonstrated impressive performance in recent years, it has encountered substantial disruptions in its SC. The Russian-Ukraine war has negatively impacted the import of raw materials for cloth manufacturing, increasing production costs. The fall of the Bangladeshi currency against the US dollar has also negatively affected the fashion industry. The COVID-19 pandemic resulted in foreign buyers canceling orders worth nearly US$ 2.9 billion, involving more than 900 million units of Bangladeshi garment products. This, in turn, led to the distress of approximately 2.27 million workers [16].

According to a Regulation of International Supply Chains (RISC) report, the pandemic introduced key challenges to Bangladesh’s fashion retail industry. These challenges included order cancellations, delayed acquisition of raw materials due to heavy reliance on China, and financial crises faced by suppliers [17]. In the meantime, other countries, including China, Vietnam, and India, have caught up with the low-cost value propositions. They are increasingly challenging the Bangladeshi fashion retail industry through their low-cost production capability. In this vein, considering these adversities, Bangladesh must seek alternative ways of maintaining competitive advantages through augmenting sustainable supply chain (SSC) management practices [18]. Combining these challenges to supply chain sustainability (SCS) and the significant repercussions of recent disruptions has prompted a systematic approach to identify, prioritize, and assess the factors influencing SCS.

Concerning the significance of sustainability, several studies have been conducted in recent years on sustainability drivers [19, 20]. The social aspects of SSC management in the apparel industry have been investigated by Köksal et al. [21] and Islam et al. [22], who provided a literature review on environmentally friendly operational practices in textile manufacturing. Paul et al. [23] explored the sustainability recovery challenges of the garment SC in the context of COVID-19 using the grey-DEMATEL approach. Reshad et al. [24] prioritized the challenges and strategies regarding the risks of managing a sustainable SC by combining the TOPSIS and VIKOR approaches. Chowdhury and Quaddus [25] investigated the interaction effect between sustainability and governance in predicting firm performance through the PLS-SEM approach.

However, there is a lack of research regarding systematic modeling of the triple bottom line of sustainability in the fashion retail industry in a developing country like Bangladesh. Also, the changing scenario in the Russian-Ukraine war period and the emerging new global SC issues have not been considered while exploring the imperatives of sustainability. However, the fashion clothing SC has several vulnerability issues that must be considered. The clothing industry discharges a high volume of waste, one of the most polluting industries in the world [26, 27]. Less than 1% of clothing waste is subject to recycling, and more than 75% is discharged into landfills worldwide [28].

Moreover, Paździor et al. [29] showed that fashion is the second-highest water-consuming industry. Because Bangladesh is one of the significant fashion cloth exporters globally, the fashion retail industry of Bangladesh needs to embrace the triple bottom line of sustainability. Therefore, considering the above research gap and the urgency of sustainability, the current research sets the below research questions (RQs):

***RQ1***: *What are the imperatives for improving SCS following the recent* SC *disruptions*?
***RQ2***: *What is the hierarchical ranking among the identified imperatives of SCS*?
***RQ3***: *How can the imperatives contribute to achieving sustainable industrial practices*?

The study followed a Pareto-based Bayesian best-worst method (BWM) approach to address the RQs to identify and model the imperatives related to an SSC. First, a thorough literature review was done to determine the primary SSC imperatives in the fashion retail industry. Secondly, Pareto analysis was performed with the experts to prioritize the SCS imperatives based on their relative importance. Finally, the Bayesian BWM approach was utilized to evaluate the significance of each of the imperatives. The employment of the integration of the Bayes theorem and the BWM method offers significant benefits over conventional multiple-criteria decision-making (MCDM) techniques. The BWM allows for a more straightforward yet deeply insightful comparison of imperatives, reducing the cognitive load on respondents and improving the reliability of the findings. The Bayesian extension incorporates uncertainty directly into the decision-making process, a reflection of real-world complexities often glossed over by other methodologies. This probabilistic approach enables a more flexible and adaptable understanding of SSC imperatives, aligning closely with the unpredictable nature of global SCs.

We selected Pareto analysis and Bayesian BWM together because they complement each other in achieving this study’s specific goals, and their order was chosen based on the specific strengths of each method. For instance, Pareto analysis helps to identify and eliminate imperatives that have minimal overall impact. By applying the 80/20 rule, it allows us to reduce the complexity of the decision-making process and focus on the most significant imperatives. The primary aim of using Pareto analysis is to streamline the list of imperatives, ensuring that only the most critical ones are carried forward. Later, Bayesian BWM utilizes the prioritized list from Pareto analysis and assigns precise weightings to the remaining imperatives. This method is particularly useful for quantifying the relative importance of each imperative with greater accuracy, based on expert opinions.

In this study, Pareto analysis was used first, followed by the Bayesian BWM. This is because Pareto analysis works like a filtering tool. It reduces the initial complexity by eliminating imperatives that have less impact on the overall goal. This ensures that the following analysis tool (which is Bayesian BWM, in this study) is focused only on the top-priority imperatives, preventing resource dilution on less critical factors. In other words, once the Pareto analysis reduces the number of imperatives, Bayesian BWM is then used to fine-tune the prioritization process of the remaining imperatives.

In summary, these methods were chosen together in this study, particularly for their complementary nature. Pareto analysis simplifies the problem by identifying the "vital few" imperatives, while Bayesian BWM is applied later and provides a structured way to determine the precise prioritization of those key imperatives. Together, they allow for both prioritization and precision in resource allocation, which is critical in a complex decision-making environment like supply chain sustainability.

This study significantly advances the discourse on SCS by meticulously identifying and prioritizing SCS imperatives across economic, social, and environmental dimensions in the context of supply chain disruptions such as wars and pandemics. This investigation is particularly novel as it is one of the initial attempts to analyze the imperatives from these three comprehensive perspectives, offering a holistic view of sustainability challenges and opportunities within supply chains. The study also distinguishes itself through methodological innovation, employing a pioneering combination of Pareto analysis and Bayesian BWM to forge an integrated model of SCS imperatives. This methodological novelty offers a fresh perspective and overcomes the limitations of traditional approaches, providing deeper insights into the prioritization of SCS strategies. Notably, the context of Bangladesh amidst the Russian-Ukraine war and post-pandemic period serves as a critical case study, filling a significant gap in the literature by delivering profound and generalizable empirical evidence on the ramifications of such global disruptions on SCS. Consequently, the findings are poised to equip supply chain practitioners and policymakers with the knowledge to construct more resilient and sustainable supply chains capable of withstanding future serious disruptions. This contribution not only enriches the theoretical landscape of SCS but also offers pragmatic guidance for enhancing supply chain resilience in developing countries, marking a considerable advancement in both the academic and practical realms of supply chain management.

The paper has been structured as follows. Section 2 presents the literature review on SCS from three bottom lines perspective. It synthesizes different studies on SCS and sheds light on the extant research gaps, followed by identifying SCS imperatives. Then, section 3 explains the methodology and justification of using a joint Pareto-Bayesian BWM approach. Sections 4 and 5 elaborate on the operationalization and results of the analysis. The discussion of the study results is presented in Section 5, along with the implications of the study from different perspectives. Finally, Section 6 provides the conclusions, including the research’s limitations.

## 2. Literature review

### 2.1 Recent studies on supply chain disruptions and sustainable supply chain

SC disruptions can be described as risks and undesirable phenomena that emerge from natural or man-made disasters such as weather turbulence, economic crisis, and the recent COVID-19 pandemic, or global wars such as the Russia-Ukraine conflict, Hamas-Israel war that affects supply chain performance [30, 31]. SC interruptions intervene in the flow of manufacturing and distribution of goods or generate complexity in the firm performance [2, 23]. Some of the expected outcomes of disruptions encompass the price spiral, reduction in global trade, value creation, and delivery of an organization [32, 33]. Recent SC disruptions, such as the Russia-Ukraine war and the COVID-19 pandemic, have exposed the vulnerability and ripple effects in the SC [34, 35]. Due to wreckage caused by the Russian war and COVID-19, there have also been chronic impacts on international trade.

The outbreak of war and pandemic have worsened the management of logistics providers and even complicated the process of upholding their contractual obligations to customers. Organizations came under intense pressure from COVID-19 and ensuing disruptions to deliver core and non-core services [36]. Most industries were significantly impacted by the COVID-19 pandemic [37, 38]. For instance, the World Trade Organization (WTO) predicted the COVID-19 pandemic might cause a 13–32% decline in global trade in 2020 [25]. The coronavirus had a significant economic impact in addition to direct and indirect value-added damages. For instance, China’s industries were considered exceptional in terms of SC segments. Still, when COVID-19 outbreaks occurred, it impacted most of these businesses’ SC networks [39]. Moreover, due to globalization, SC disruptions have affected both large and small and medium-sized enterprises (SMEs) in developed and developing economies.

Considering the recent disruptive SC events, organizations and policymakers have focused on devising solutions to meet customer demands after such disruption. Predicting the effects would be more challenging because every pandemic is unique. Even if the COVID-19 outbreak in emerging countries only affected a relatively small number of enterprises and was prevented by segregation and community lockdowns, exceptionally high unemployment rates and uncertain economic situations have created challenges for social sustainability [40]. In the current post-pandemic period, the SC literature must look at the possible intervention programs that can be put into practice to maintain a regular flow of products and services and to prevent the collapse of supply chains in the event of any future disasters. The SCs’ resilience and viability must be augmented through precautionary measures [36, 41]. Now, companies are concentrating on formulating risk management strategies that could mitigate the impacts of such disruptions and make the SC resilient and viable in any future pandemic or war-like disruptions [42]. As a proactive measure for improving disaster readiness, the widely used concept of sustainable SC management is a strategy for managing SC operations toward achieving environmental, economic, and social sustainability for the organization and the broader environment.

Sustainable Supply Chain (SSC) management entails managing and sustaining supplier relationships with economic, social, and environmental concerns. Organizations can benefit by including environmental principles in SC initiatives [41]. Implementing SSC can help a company with marketing, raise the prominence of its brand, and increase its popularity. Cost reductions are another essential advantage of implementing SSC management [42]. Since sustainability research is still insufficiently built, developed, and applied, many businesses in prosperous economies cannot successfully embrace and progress toward sustainability [41, 43]. However, as emerging economies also encountered SC disruptions, managing sustainability issues is equally essential due to the uncertainty and challenges associated with SSC management.

### 2.2 Imperatives of sustainable supply chain in the context of recent disruptions

Increased awareness of sustainability dimensions is necessary to navigate SC disruptions, such as wars and pandemics, by addressing social and environmental concerns towards creating final products or services [44]. The organization’s internal and external variables may influence how sustainable SC management is implemented. These elements can significantly affect the adoption of SSC and improve performance and reactivity [45]. The Famental aspects of sustainability from the social, economic, and environmental viewpoints enhance organizational performance. Additionally, it guarantees risk management and economic success [46]. Investigating the elements of sustainability is necessary, mainly when dealing with war and pandemic-like effects on supply chains. These elements support businesses in enhancing sustainability initiatives and increasing sustainability’s overall efficacy.

To identify the key imperatives enhancing SCS within the specific context of Bangladesh, an extensive research endeavor was undertaken. This effort encompassed a comprehensive search for literature across well-established databases such as Scopus, ScienceDirect, and Google Scholar, published between 2015–2023. The research was specifically geared toward exploring a range of pivotal terms, including "Supply Chain Sustainability", "Emerging Economy", "Imperatives/Factors/Drivers", and "Sustainable Development", among others. This meticulous examination yielded a compilation of 16 distinct imperatives to the successful implementation of SCS. Table 1 shows the initially identified imperatives after reviewing the literature.

**Table 1:** The initially identified imperatives of supply chain sustainability with sources.

No.	Imperatives of SCS	Sources
01	Developing strategic SC interventions to ameliorate the impact of disruptive events	[47]
02	Increasing the application of big data analytics (BDA)	[48]
03	Understanding the customer awareness of sustainable products	[49]
04	Digitizing the SC	[50]
05	Improving the collaboration among SC stakeholders	[36]
06	Exploding the use of robotics in logistics and inventory system	[51]
07	Development of intertwined supply network (ISN)	[51]
08	Increasing the use of the Internet of Things (IoT) in the SC activities	[52]
09	Integrating cloud manufacturing technologies	[53]
10	Prepare for the rebound	[47]
11	Ensuring compliance with the health and safety legislations across the SC	[41]
12	Increasing the localized production capability to buffer disruptions	[54]
13	Deploying blockchain technology in SC management	[55]
14	Increasing the application of circular manufacturing	[2]
15	Strategic planning for sustainability	[56]
16	Improving the public-private relationships	[57]

### 2.3 Study context, research gap, and contributions

This study examines the SSC imperatives that will assist the fashion retail industries in Bangladesh in developing essential policies for ensuring long-term sustainability during disruptive events like COVID-19 and the Russian invasion of Ukraine. One of Bangladesh’s top sectors for remittance earnings is the fashion retail industry, which contributes to the country from multiple directions, boosting economic growth, poverty mitigation, women empowerment, job opportunities, etc. While the fashion retail industry has demonstrated impressive performance in recent years, it has encountered substantial disruptions in its SC. The Russian invasion of Ukraine has disrupted this sector recently and imposed unprecedented impacts.

Due to this situation, foreign buyers have canceled their orders, and many small factories are shut down. As per the report of Bangladesh Bank, in April, the country’s fashion retail exports observed a significant decline of 15.48%, amounting to $3.32 billion compared to April 2022 [58]. According to a RISC report, the pandemic introduced vital challenges to the fashion retail industry in Bangladesh. These challenges included order cancellations, delayed acquisition of raw materials due to heavy reliance on China, and financial crises faced by suppliers [17]. The combination of these challenges to the SCS, coupled with the significant repercussions of recent disruptions, has prompted the need for a systematic approach to identify, prioritize, and assess the imperatives influencing SCS.

Researchers have previously examined SC issues, obstacles, information sharing, convergence, networks, and supplier engagement. Even though literature has drawn attention to SC issues, a complete examination of the critical elements required to enhance an SSC in the context of chronic disruptive events such as global wars and COVID-19 has not yet been carried out. The literature on sustainability drivers is fragmented regarding SC disruptions. Most study works have only examined drivers and obstacles towards sustainability [41]. Only a few studies have examined their dependence on sustainability drivers and their relationships. Therefore, this research fills this gap and investigates the sustainability imperatives and their interrelationships so businesses can develop creative suggestions for formulating sustainable and competitive supply chains to succeed under disruptions.

This study offers several substantial contributions to the existing literature. First, it enhances scholarly discourse by meticulously examining and analyzing the imperatives that strengthen SCS during disruptive events, such as pandemics and geopolitical conflicts, specifically within the fashion retail industry of Bangladesh. This study underscores the critical role of SCS during such challenging times. Secondly, the study marks a significant advancement by prioritizing these imperatives. It employs a novel approach by integrating advanced Pareto analysis with Bayesian BWM theory, focusing on primary aspects like social, economic, and environmental criteria. This represents a pioneering effort in amalgamating Bayesian BWM, the context of an emerging economy, and imperatives enhancing SCS within a unified framework. Furthermore, the study provides empirical evidence supporting the effectiveness of these strategies, offering valuable guidance for decision-makers. This guidance is particularly useful for proactive decision-making. The approach is also geared towards maintaining sustainable industrial practices in the face of disruptive events. Lastly, the implications of this study for Sustainable Development Goals (SDGs) are profound, notably impacting SDG 1 (No Poverty), SDG 8 (Decent Work and Economic Growth), SDG 9 (Industry, Innovation, and Infrastructure), SDG 12 (Responsible Consumption and Production), SDG 15 (Life on Land), and SDG 16 (Peace, Justice, and Strong Institutions), among others. This research not only adds to academic knowledge but also provides practical solutions and strategies for sustainability in this challenging time.

## 3. Method

### 3.1 Survey design

The study employs a structured, innovative five-phase methodology, meticulously designed to address SSC imperatives in the fashion retail industry of emerging economies, particularly against the backdrop of global disruptions. This approach begins with an exhaustive literature review, which is crucial for establishing a comprehensive foundation by identifying SSC imperatives and integrating a broad array of scholarly insights. This step goes beyond traditional methods by ensuring a deep, contextual understanding of the SSC field, tailored to the unique needs of emerging markets. Distinctively, the methodology engages Bangladeshi fashion retail industry experts in its second phase, ensuring the findings are not only relevant but also infused with local expertise, a step often overlooked by generic approaches.

A combination of Pareto analysis and the Bayesian BWM was used in this study to efficiently prioritize and allocate resources across the identified supply chain sustainability imperatives. Pareto analysis was applied first to filter the imperatives, focusing on the top 20% that contribute to roughly 80% of the overall impact. This step simplified the decision-making process by removing less critical imperatives. After applying Pareto analysis, Bayesian BWM was used to assign precise weightings to the remaining imperatives, providing a detailed ranking based on expert input. Therefore, in short, these two methods were used in this order to ensure the accuracy and practicality of the prioritization process of the imperatives and their subsequent assessment. Due to the high efficiency of this methodological approach, a similar approach was adopted in several previous multiple-criteria decision-making (MCDM)-based studies [2, 59].

**Fig 1:**
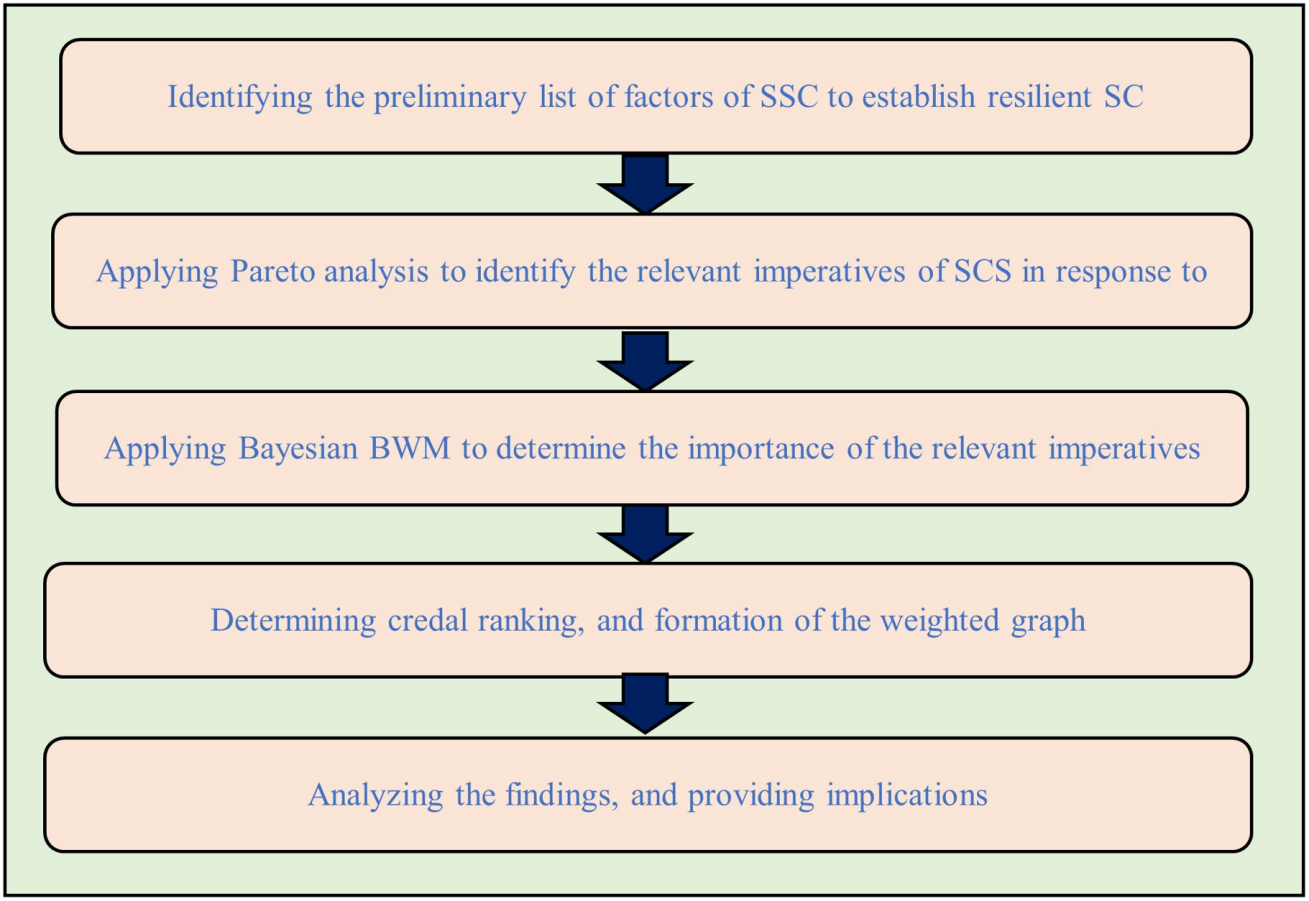
Necessary steps for the research framework.

After applying Pareto analysis, this study later combines the Bayes theorem with the BWM method to ensure a robust, unambiguous, and probabilistic evaluation of each imperative. Bayesian BWM is chosen for its superior ability to handle uncertainty and complexity, offering a nuanced analysis that traditional deterministic methods, such as AHP, TOPSIS, or VIKOR cannot match [59]. This advanced analytical technique allows for a more adaptable and precise understanding of SSC strategies, providing a significant edge in navigating the volatile nature of global supply chains. By detailing the unique context of Bangladesh and leveraging advanced analytical techniques, this methodology not only advances theoretical understanding but also offers practical, evidence-based recommendations for SSC management. The methodology, illustrated in Fig 1 for clarity, aligns with the objective of advancing SSC management by delivering novel, evidence-based insights tailored to the unique context of Bangladesh, thereby offering a methodologically sound pathway to achieve the research objectives.

A panel of 24 experts from various backgrounds, including the fashion retail industry, supply chain management, and academia, was carefully chosen to identify the pertinent imperatives for establishing sustainability. The process ensured confidentiality by not disclosing the experts’ names. The selection criteria were meticulously crafted, encompassing factors such as job title, years of experience, expertise in the fashion retail industry, sustainability, and supply chain management, among other relevant qualifications. This method, termed "purposive or deliberate sampling," involves a purposeful selection based on the researcher’s assessment instead of a random approach [60–62]. While subjectivity is a limitation, deliberate sampling facilitates the collection of high-quality expert insights within resource constraints [63, 64].

Experts were contacted via email using a Google form and invited to share their opinions. The questionnaire (See Appendix A in S1 File) incorporated key imperatives from literature resources. Experts were prompted to answer queries such as their organization type, job responsibilities, and years of experience and to contribute relevant imperatives to aid fashion retail industries in Bangladesh in crafting policies for long-term sustainability. Experts were also given the liberty to modify the provided list of imperatives. Throughout the survey, experts removed two imperatives (*Strategic planning for sustainability* and *Prepare for the rebound*) and introduced four additional ones. The newly added imperatives are *’Using Artificial Intelligence (AI) in manufacturing systems*, *Availability of Government support schemes to promote sustainability*, *Enhancing the resilience of transportation and logistics system* and *Enabling scattered supplier management system*. The revised list of imperatives after expert validation has been presented in Table 2.

**Table 2:** The revised list of factors after expert validation.

Imperatives of SSC	Code	Source
Developing strategic SC interventions to ameliorate the impact of disruptive events	I1	[47]
Increasing the application of big data analytics (BDA)	I2	[48]
Understanding the customer awareness of sustainable products	I3	[49]
Digitizing the SC	I4	[50]
Improving the collaboration among SC stakeholders	I5	[36]
Exploding the use of robotics in logistics and inventory system	I6	[51]
Development of intertwined supply network (ISN)	I7	[51]
Increasing the use of the Internet of Things (IoT) in the SC activities	I8	[52]
Integrating cloud manufacturing technologies	I9	[53]
Ensuring compliance with the health and safety legislations across the SC	I10	[41]
Increasing the localized production capability to buffer disruptions	I11	[54]
Deploying blockchain technology in SC management	I12	[55]
Increasing the application of circular manufacturing	I13	[65]
Improving the public-private relationships	I14	[57]
Using Artificial Intelligence (AI) in manufacturing system	I15	Expert feedback
Availability of government support schemes to promote sustainability	I16	Expert feedback
Enhancing the resilience of transportation and logistics system	I17	Expert feedback
Enabling a scattered supplier management system	I18	Expert feedback

With a comprehensive consideration of literature and expert inputs, 18 SCS imperatives were selected for examination in an emerging economy like Bangladesh. A subsequent questionnaire (Appendix A in S1 File) was designed based on these imperatives and distributed digitally to the same experts for use in the Pareto analysis. Experts assessed the priority weights of SCS imperatives using a 5-point Likert scale. The cumulative scores were evaluated for further investigation, focusing on variables that accounted for approximately 80% of the total score [66]. Through Pareto analysis, the relevant imperatives were refined, leading to the subsequent implementation of the BWM survey. The BWM survey was constructed considering three sustainability dimensions: economic, social, and environmental.

### 3.2 Data collection

During the fourth phase of our study, we executed a BWM survey. To ensure a comprehensive pool of participants, we utilized multiple approaches, including emailing fashion retail industry managers and disseminating the study through social media platforms such as Facebook and LinkedIn. We also made use of a Google form to facilitate the survey process. In the initial stage of the survey, experts were requested to identify the best and worst imperatives of SCS. Subsequently, they were guided to construct two sets of comparison vectors, Best-to-Others and Others-to-Worst, individually for each of the three sustainability perspectives–economic, social, and environmental. The data collection spanned from July 1, 2022, to January 31, 2023. Over this period, we initially invited 50 experts to take part in our study. Twenty-four of them responded (48% response rate) and participated in the factor validation and Pareto analysis. Those 24 experts were invited again for the Bayesian BWM analysis. Only 8 out of those 24 experts responded (33.33% response rate) in this stage.

This study did not involve any human subject, minor, animal, medical specimen, or medical information. The willing and informed adult industry experts provided their professional opinion/feedback (which contained no medical information) via online Google forms that we sent them via email. The email was only sent to those experts who verbally agreed to participate in the study over the phone. All verbal agreements were made in the presence of at least two of the research project members. The collected data from the experts were processed anonymously to ensure an unbiased study.

A comprehensive profile of the respondents can be found in Appendix C in S1 File. The development of Best-to-Others and Others-to-Worst vectors are shown in Appendix B in S1 File. These tables provide a transparent representation of the construction process. With these comparison vectors in place, we applied the Bayesian-BWM to derive meaningful insights from the collected data.

### 3.3 Bayesian best-worst method

In the fifth phase of our study, the Bayesian BWM was employed to establish the priority importance of the SCS imperatives. This innovative approach, stemming from the conventional BWM, introduces a novel perspective to group decision-making. In contrast to the precise values the traditional BWM assigns, Bayesian-BWM offers a framework for determining the comprehensive criteria weights [67]. Notably, one of its distinctive features lies in its capacity to accommodate multiple experts or decision-makers, evaluating their aggregated consequences through a probabilistic lens [68].

A key aspect of Bayesian-BWM is its ability to address the limitations of using averages, which can be sensitive to outliers and treat each expert’s opinion equally. When dealing with numerous decision-makers, relying solely on the average operator can lead to substantial inaccuracies and misleading information. To counteract this, Bayesian-BWM introduces a more robust methodology that leverages probabilistic reasoning. Herein, we outline our study’s primary procedures employed in the Bayesian-BWM framework.

***Step 1***: ***Deciding on a set of standards judged by the experts*.**
  *C* = {*c*_1_, *c*_2_, …, *c*_*n*_}, where there are n total criteria.
***Step 2*: *Identifying the best* (*C***_***B***_**) *and the worst* (*C***_***w***_**) *criteria from C*.**
  In this step, each expert is requested to identify only the best and worst criteria from the set specified as *C*. Here, the best standards denote the most essential imperative for ensuring sustainability, whereas the worst one is the least necessary for all experts.
***Step 3***: ***Comparing the best criterion in pairs with the other standards*.**
  Here, the experts develop a comparison vector in pairs between the best and others. Using 1 to 9 numbers, the experts create the comparison matrix. If any expert gives 9, it means the best criterion possesses more importance than others, and the value of 1 refers to equally important. The Best-to-Others vector is represented as *A*_*B*_ = (*a*_*B*1_, *a*_*B*2_, …, *a*_*Bn*_). Here, *a*_*Bj*_ indicates the relative importance of the best over others *c*_*j*_ ∈ *C*.
***Step 4***: ***Comparing the worst criterion in pairs with the other standards*.**
  Similarly, each expert uses a scale of "1 to 9" to indicate how important the other criteria are compared to the worst. It is used to denote the next Others-to-Worst vector. The relative relevance of the other criteria *c*_*j*_ ∈ *C* over the worst one is shown here by the symbol *a*_*wj*_
***Step 5***: ***Calculate the total weight and the individual ideal weight*.**
  Here, each *z*^*k*^ denotes the optimal weight, and the z* is the aggregated optimal weight determined, where *K* represents the experts’ number in total.
$$\left.A_{B}^{k}\right|z^{k}\sim \text{multinomial}\;\left(\frac{1}{Z^{k}}\right)$$ (1)
$$\left.A_{W}^{k}\right|z^{k}\sim \text{multinomial}\;\left(\frac{1}{Z^{k}}\right)$$ (2)
$$z^{k}\left|z^{*}\right.\sim Dir\left(\gamma \times z^{*}\right)$$ (3)
$$\gamma \sim \text{gamma}\left(0.1,0.1\right)$$ (4)
$$z^{*}\sim Dir\left(1\right)$$ (5)

Here, *multinomial and Dir refer to* the multinomial distribution *and* Dirichlet distribution, respectively, while γ is the concentration factor. Eqs (1)–(5) do not form a closed-loop model, so Markov-chain Monte Carlo (MCMC) is required. The Bayesian-BWM is applied using Just Another Gibb Sampler (JAGS), one of the best available feasible languages.

The credal ranking was applied to determine the priority weights of the imperatives of SCS and the degree to which one criterion is favored over another. The following definitions of credal order and ranking will help to estimate the priority weights and the degree of preference [69].

*Definition 1*: *A credal ordering O for a pair of criteria* *c*_*i*_ *and c*_*j*_, *can be mentioned as*:

$$O=\left(c_{i},c_{j,}R,d\right)$$ (6)

Here, R represents the relationship between the criteria, and d represents the degree of confidence in the superiority of the requirements.

*Definition 2*: A succession of credal orderings is used to express the credal rating. For a set of criteria, *C* = {*c*_1_, *c*_2_, …, *c*_*n*_} it comprises all pairs (*c*, *c*_*j*_) for all *c*_*i*_, *c*_*i*_ ∈ *C*. If the sample size is *M*, the confidence that *c*_*i*_ being superior to *c*_*j*_ can be computed using the following equation:

$$P\left(c_{i}>c_{j}\right)=\frac{1}{M}\sum_{m=1}^{M}\;I\left(z_{i}^{agg_{m}}>z_{j}^{agg_{m}}\right)$$ (7)

## 4. Results

**Fig 2:**
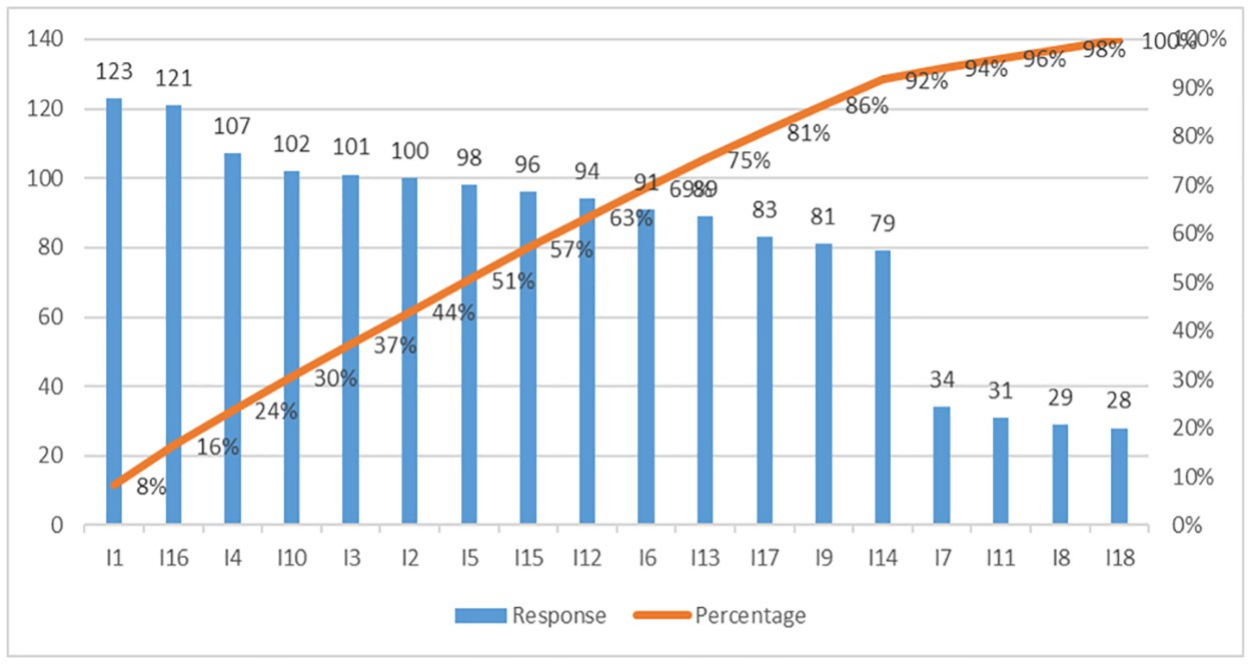
Pareto chart for the imperatives of SCS.

At this phase of the study, the outcomes derived from employing the Pareto-based Bayesian-BWM framework have been showcased to comprehend the varying significance of the key imperatives of SCS in addressing the repercussions of recent disruptions in the SC, particularly within the context of the fashion retail industry in Bangladesh. It’s worth noting that the study identified eighteen distinct imperatives of SCS due to a combination of literature analysis and expert input. However, it’s essential to acknowledge that not all these imperatives hold the same relevance level within this study’s specific scope. Their pertinence is influenced by factors like the country’s setting, economic viewpoint, cultural nuances, and social considerations. To finally select these SCS imperatives, this study adopts the Pareto analysis technique. Experts have been engaged to evaluate these weights, utilizing a 5-point Likert scale. Fig 2 shows the imperatives and the cumulative percentage score.

Twelve of the eighteen imperatives are considered for further analysis depending on the cumulative percentage score, which collectively encompasses 80% of the overall score. Twelve most significant imperatives to SCS are: *"Developing strategic SC interventions to ameliorate the impact of disruptive events(I1)"*, *"Digitizing the SC (I4)"*, *"Using artificial intelligence (AI) in manufacturing system (I15)"*, *"Understanding the customer awareness for the sustainable products (I3)"*, *"Ensuring compliance with the health and safety legislations across the SC (I10)"*, *"Increasing the application of big data analytics (BDA) (I2)"*, *"Improving the collaboration among SC stakeholders (I5)"*, *"Exploding use of robotics in logistics and inventory system (I6)"*, *"Availability of government support schemes to promote sustainability (I16)"*, *"Deploying blockchain technology in SC management (I12)*, *"Increasing the application of circular manufacturing (I13)"*, *"Enhancing the resilience of transportation and logistics system (I17)"*. The finally selected twelve most significant imperatives after the Pareto Analysis are shown in Table 3.

**Table 3:** Brief description of the finally selected imperatives after Pareto analysis.

Code	Imperatives of SCS	Description	Sources
I1	Developing strategic SC interventions to ameliorate the impact of disruptive events	Create hypothetical situations based on different disruptive events to understand better the potential effects and difficulties associated with each scenario. Closer links with suppliers can help reduce SC’s vulnerability due to various disruptions.	[47]
I4	Digitizing the SC	The two main advantages of digitizing a SC’s operation are speed and cost. Digitizing processes will increase visibility and offer real-time access to the SC, allowing those involved to establish complete control.	[50]
I3	Understanding the customer awareness of sustainable products	Well-planned customer awareness educates us to utilize sustainable products and services. Sustainable products give the benefit of long-term economic benefits, health advantages, environmental preservation, and a higher quality of life.	[49]
I2	Increasing the application of big data analytics (BDA)	Organizations can use advanced and real-time data analytics to minimize lead times and needless transportation. They can send the right offer to the appropriate person at the proper time.	[48]
I5	Improving the collaboration among SC stakeholders	Collaborative planning with the SC parties improves visibility, traceability, and amount of data sharing. It also helps increase secured logistics capacity on more favorable terms.	[36]
I6	Exploding the use of robotics in logistics and inventory system	This has made the SC more autonomous. The use of robots in manufacturing ensures safety and improves productivity.	[51]
I13	Increasing the application of circular manufacturing	By increasing its agility, economy, and ability to adapt to shifting market conditions, circular manufacturing can change the manufacturing sector completely.	[65]
I15	Using Artificial Intelligence (AI) in manufacturing systems	AI can completely transform the manufacturing sector by boosting output, cutting expenses, and raising product quality. Manufacturers can maintain their leadership position in the market and obtain a competitive edge by utilizing AI technologies.	Expert opinion
I10	Ensuring compliance with the health and safety legislations across the SC	It is possible to lower risks and avoid injury and create a more resilient, moral, and ecologically conscious SC that is advantageous to all parties involved by including health and safety considerations into SCS practices.	[41]
I16	Availability of government support schemes to promote sustainability	Usually, the goals of these programs are to promote sustainable development, economic uplifting, and encourage environmentally friendly behavior.	Expert opinion
I12	Deploying blockchain technology in SC management	Give suppliers the data privacy they need and buyers the visibility they want. This enables smart contact and ensures authentic transactions, increasing reliability and transparency.	[51]
I17	Enhancing the resilience of transportation and logistics system	Adopt ecologically conscious and sustainable logistical methods. This includes looking into alternative energy sources for transportation, utilizing fuel-efficient cars, and streamlining routes to cut pollution. While reducing adverse effects on the environment and society, it also guarantees the efficient movement of goods.	Expert opinion

As per the initial step of the BWM approach, the twelve imperatives of SCS, chosen through the Pareto analysis, are considered alternatives for assessment within the context of the fashion retail industry. Later, in this study, we categorized the 12 imperatives into three sub-categories (social, environmental, and economic), which were carried out following expert feedback, especially in order to align this study with the three widely accepted pillars of sustainability (social, environmental, and economic). When the survey form was distributed among the participating experts to validate and classify the imperatives, the experts were also asked to vote on which category each imperative should belong to, and the final classification was determined through majority voting. This method ensured that the categorization was both grounded on expert insights and properly aligned with theoretical principles of ‘sustainability’. For each of the three sustainability dimensions, as detailed in Appendix B in S1 File, experts individually designate the "Best" and "Worst" alternatives. Subsequently, the "Best-to-Others" and "Others-to-Worst" vectors are formulated using these expert inputs. These vectors, about the three dimensions, are presented in Appendix B in S1 File.

Employing these vectors, a Bayesian-BWM solver implemented in MATLAB is employed to derive the ultimate weights for the key imperatives of SCS. These average weights are computed for each sustainability dimension using the two vectors. Table 3 lists the final consequences for the key SCS imperatives within the economic, social, and environmental dimensions, respectively. It is evident from Table 4 that "Availability of government support schemes to promote sustainability (I16)" holds the highest priority, boasting a weight of 0.4569. Conversely, "Increasing the application of big data analytics (BDA) (I2)" is assigned the lowest priority with a weight of 0.1072 among the four SCS imperatives in the economic dimension. "Developing strategic SC interventions to ameliorate the impact of disruptive events (I1)" has garnered the foremost priority, boasting a weight of 0.4485. Conversely, among the four SCS imperatives of social dimension, "Improving the collaboration among SC stakeholders (I5)" is ascribed to the lowest priority, carrying a weight of 0.1365. Conclusively, Table 4 outlines the average weights of imperatives from the environmental dimension. Notably, "Understanding the customer awareness of sustainable products (I3)" emerges as the topmost priority, carrying a weight of 0.3026. On the other hand, among the collection of four key imperatives of SCS in the environmental dimension, " Enhancing the resilience of transportation and logistics system (I17)" is situated at the lowest rung of priority, holding a weight of 0.1791.

**Table 4:** Average weight and global weight of the imperatives of SCS.

Criteria (sub-catagories)	Criteria weight	Imperatives of SCS	Code	Average weight	Global weight
Economic	0.3738	Digitizing the SC	I4	0.2734	0.1022
		Increasing the application of big data analytics (BDA)	I2	0.1072	0.0401
		Availability of government support schemes to promote sustainability	I16	0.4569	0.1708
		Deploying blockchain technology in SC management	I12	0.1625	0.0607
Social	0.3676	Developing strategic SC interventions to ameliorate the impact of disruptive events	I1	0.4485	0.1649
		Improving the collaboration among SC stakeholders	I5	0.1365	0.0502
		Ensuring compliance with the health and safety legislations across the SC	I10	0.2501	0.0919
		Increasing the application of circular manufacturing	I13	0.1649	0.0606
Environmental	0.2586	Understanding the customer awareness of sustainable products	I3	0.3026	0.0783
		Using Artificial Intelligence (AI) in manufacturing system	I15	0.2571	0.0665
		Exploding the use of robotics in logistics and inventory system	I6	0.2576	0.0666
		Enhancing the resilience of transportation and logistics system	I17	0.1791	0.0463

**Fig 3:**
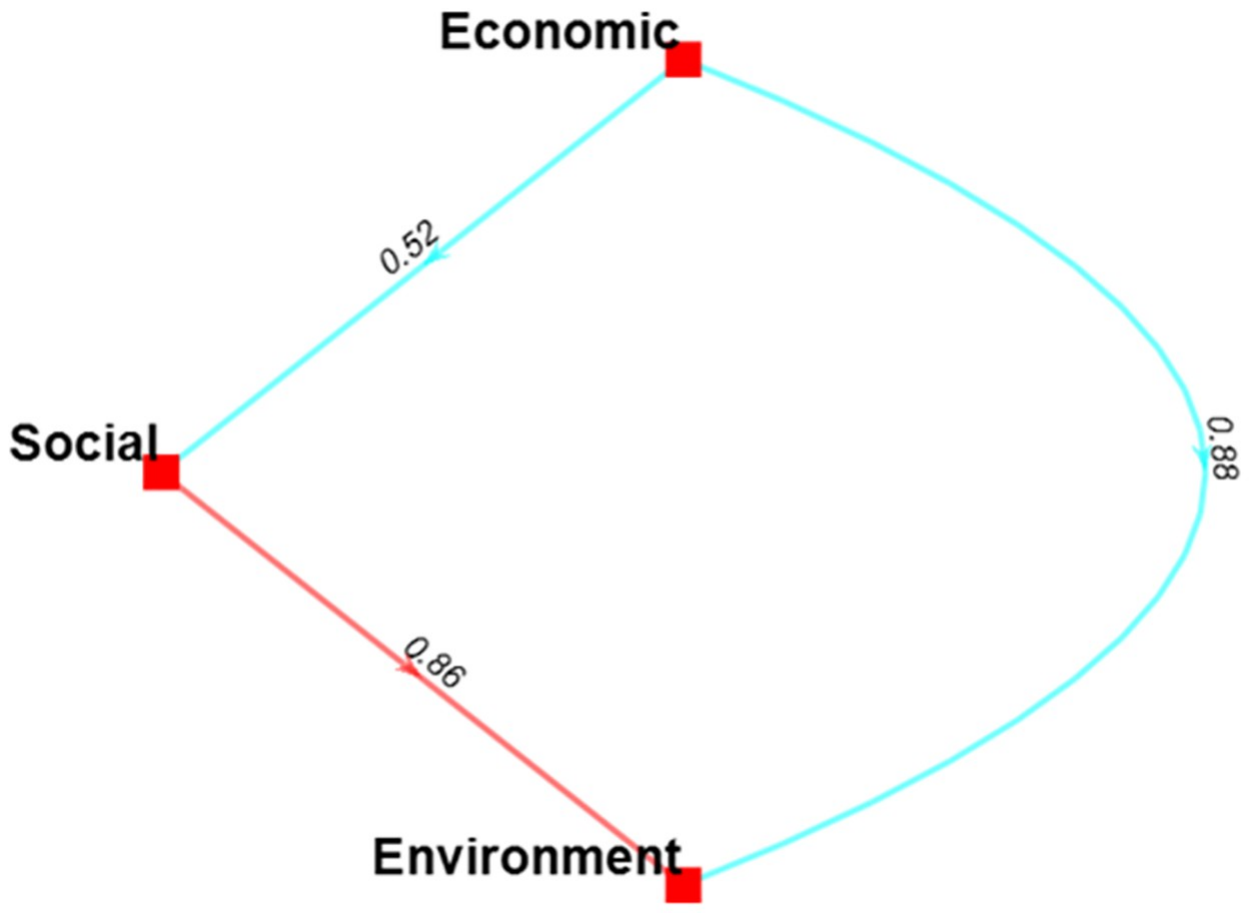
Hierarchical credal ranking of the main criteria of SCS.

A notable outcome of the Bayesian BWM methodology is the creation of a credal ranking, depicted through a weight-directed graph. This graph effectively illustrates the interconnectedness between pairs of key imperatives within SCS. To calculate the confidence of each credal ranking, a Bayesian test is developed as opposed to the conventional method, which only employs two figures to evaluate confidence superiority. In Fig 3., it portrays the confidence level associated with selecting one main criterion over another. The economic criteria are more important than the other two by having a confidence of 0.52 between the social criteria. As environmental criteria stand last, social criteria are the second most important criterion, containing a confidence of 0.86 compared to the environmental criteria.

**Fig 4:**
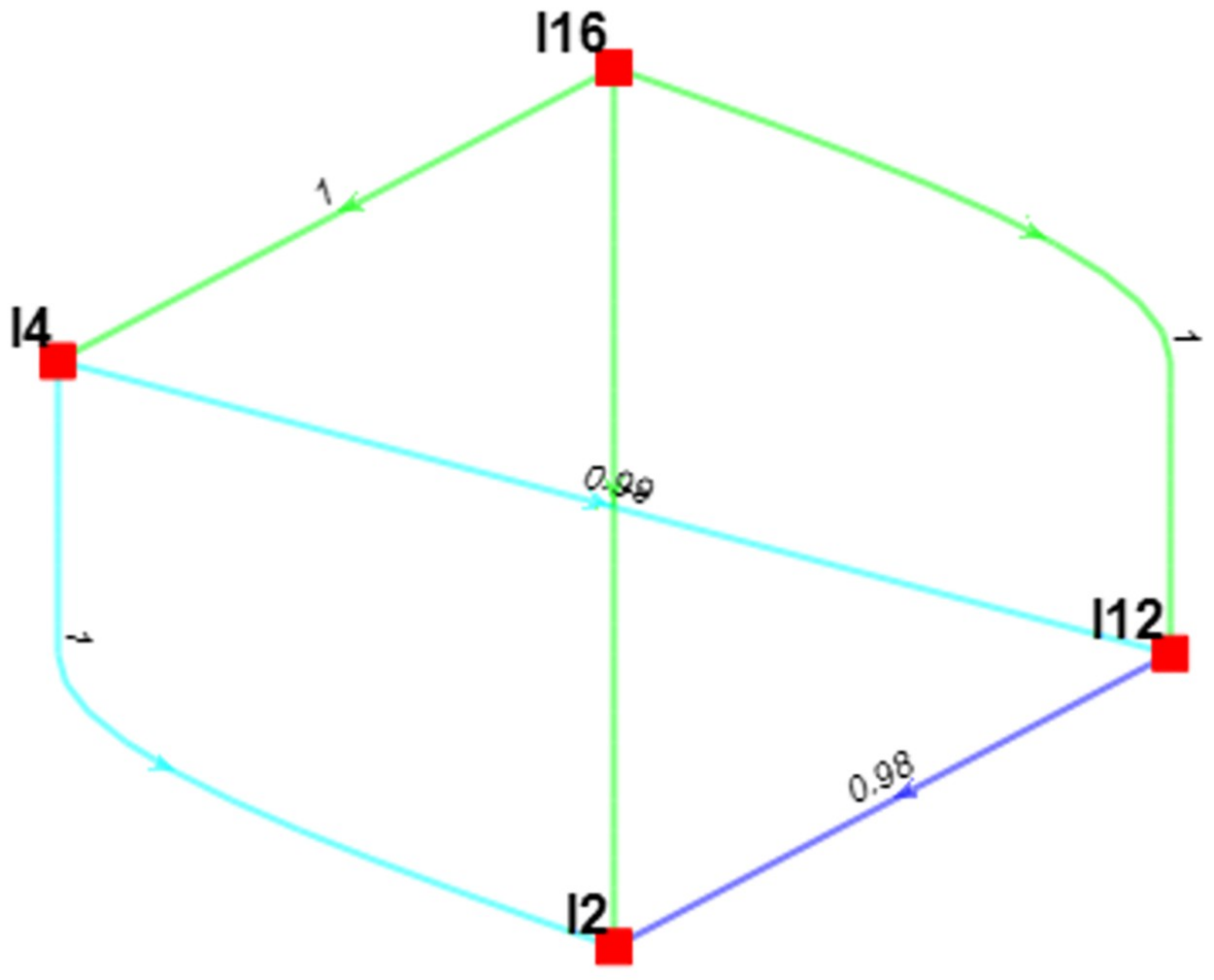
Hierarchical credal ranking of the key imperatives of SCS from an economic perspective.

Fig 4. depicts that, from an economic standpoint, the credal ranking underscores that "Availability of government support schemes to promote sustainability (I16)" stands as the most pivotal imperative of SCS, designed to mitigate the repercussions of recent SC disruptions. It holds a confidence level of 1 compared to "Digitizing the SC (I4)", which declares that government support is much more crucial for SCS. "Digitizing the SC (I4)" contains a confidence of 0.99 compared to "Deploying blockchain technology in SC management (I12)". So, based on creedal ranking in the economic criteria, "Increasing the application of big data analytics (BDA) (I2)" stands last.

The graphical representation in Fig 4. also highlights that, for each pair of imperatives, there exists a substantial level of confidence in selecting one imperative over the other, with confidence scores generally exceeding 0.9.

**Fig 5:**
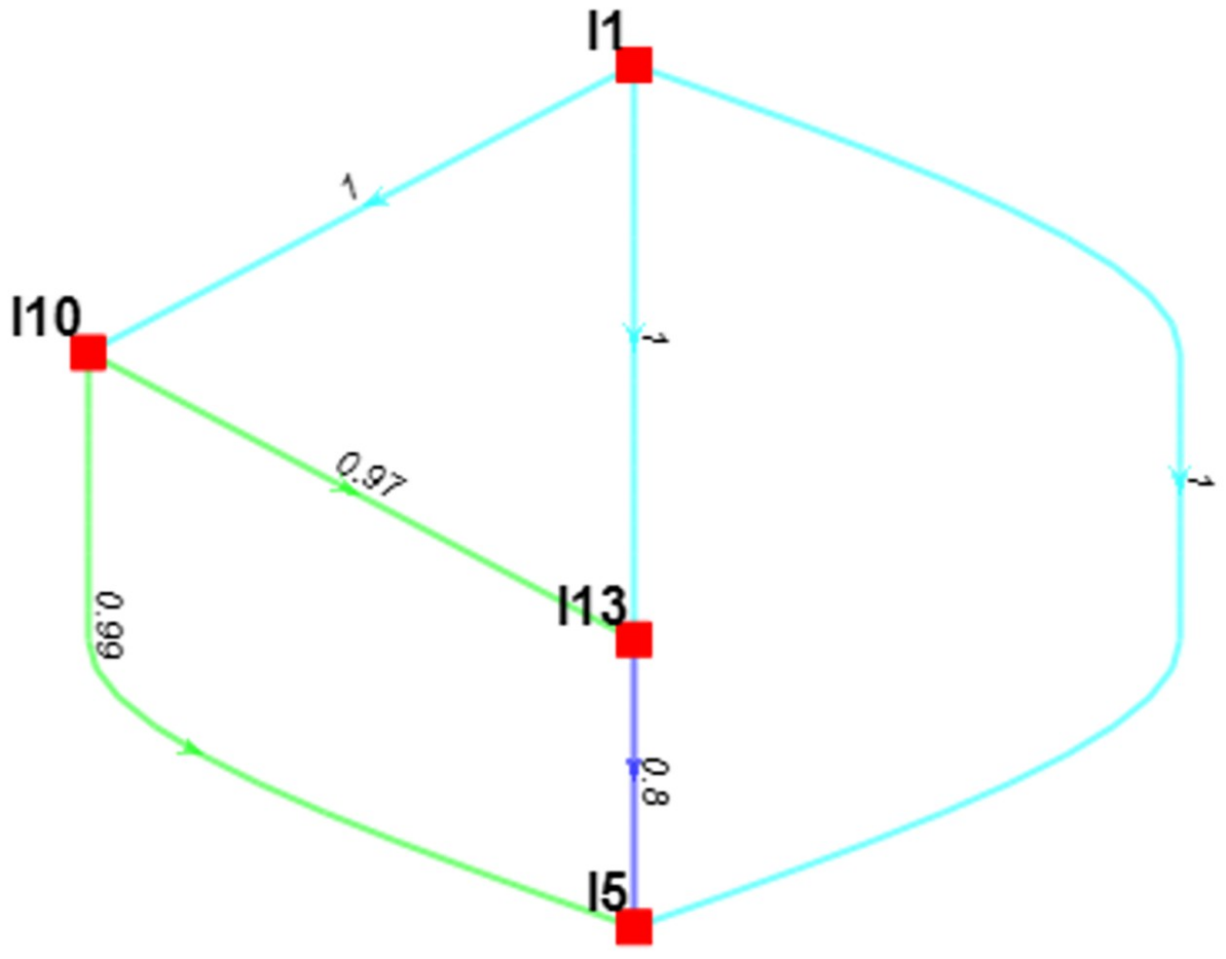
Hierarchical credal ranking of the key imperatives of SCS from a social perspective.

From a social standpoint in Fig 5, credal ranking shows that "Developing strategic SC interventions to ameliorate the impact of disruptive events (I1)" is found to be the most significant imperative of SCS to cope with the impacts of recent SC disruptions, with a confidence of 1 against "Ensuring compliance with the health and safety legislations across the SC (I10)", with a confidence of 0.97 against " Increasing the application of circular manufacturing (I13) & with a confidence of 0.8 compared to "Improving the collaboration among SC stakeholders (I5)". Fig 5. also demonstrates that for each pair of SCS imperatives, the confidence in choosing one over the other is relatively strong (above 0.8).

**Fig 6:**
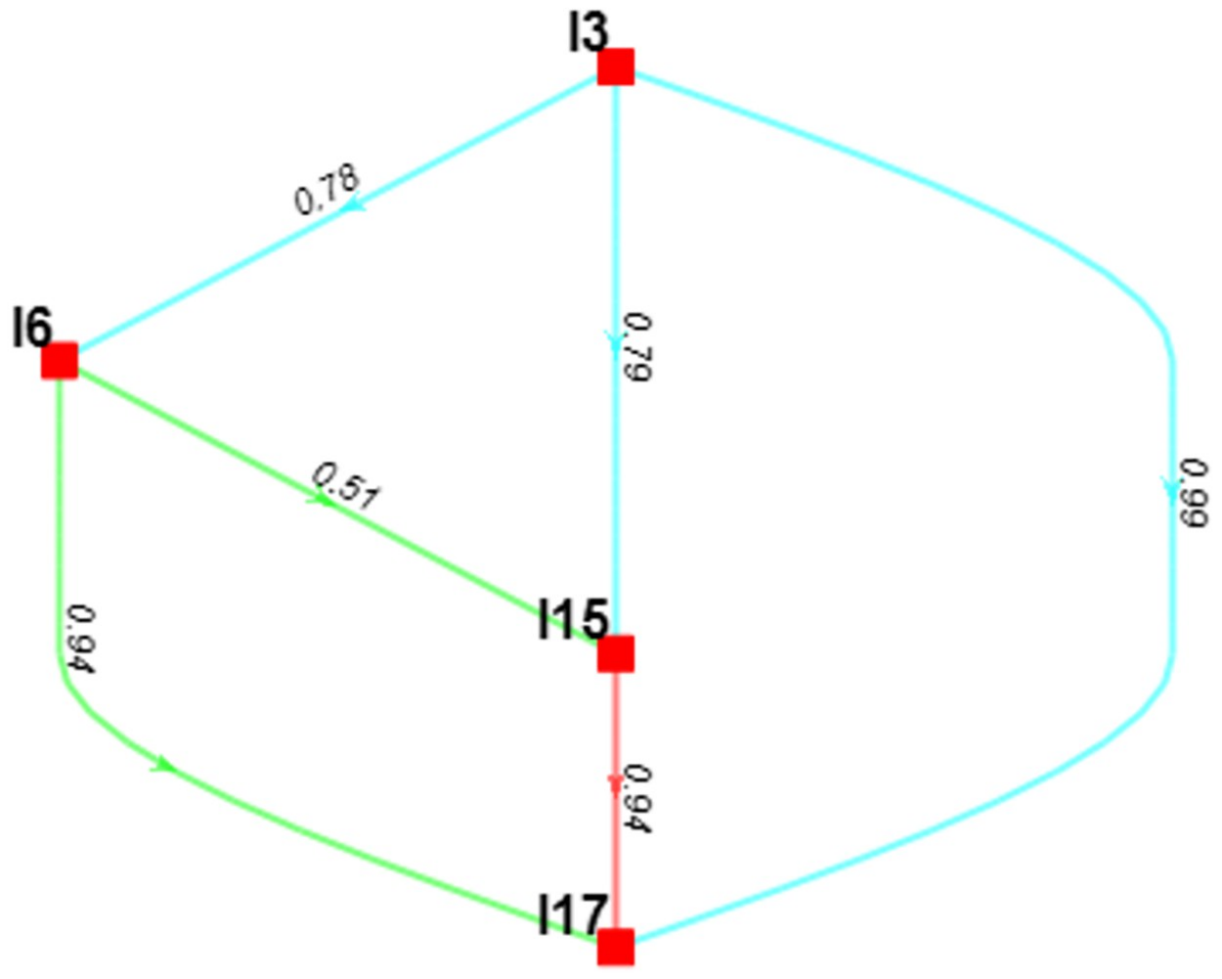
Hierarchical credal ranking of the key imperatives from an environmental perspective.

In Fig 6. examined from the environmental dimension, the credal ranking elucidates that "Understanding the customer awareness of sustainable products (I3)" emerges as the paramount imperative of SCS, essential for surmounting the challenges posed by recent disruptions in the SC. This imperative is supported by a confidence level of 0.76 in comparison to "Exploding the use of robotics in logistics and inventory system (I6)", which tells that customer awareness of sustainable products helps to increase SCS during disruptive periods like COVID-19 and geopolitical war. Additionally, it maintains a confidence of 0.52 against "Using Artificial Intelligence (AI) in manufacturing system (I15)". Therefore, "Enhancing the resilience of transportation and logistics system (I17)" stands last, so "Using Artificial Intelligence (AI) in manufacturing system (I15)" contains a confidence of 0.94 against it. Fig 6. also emphasizes that the confidence level in opting for one SCS imperative over another is notably strong, with confidence scores generally surpassing 0.5 for each pair of imperatives.

**Fig 7:**
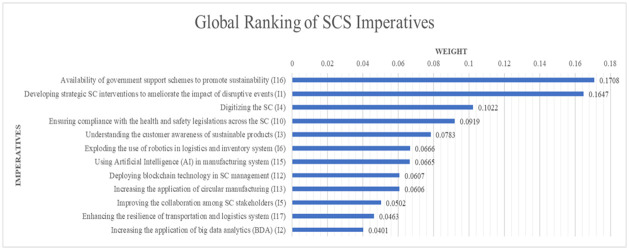
Global ranking of SCS imperatives.

Fig 7. shows the global ranking of the SCS imperatives. The global ranking is calculated by multiplying the individual main criteria weight and the average weight of the imperatives. The vectors about the three main criteria to get the main criteria weight are presented in Appendix B in S1 File. The study result shows that the "Availability of government support schemes to promote sustainability (I16)" is the most important imperative, containing a global weight of 0.1708, and Increasing the application of big data analytics (BDA) (I2) is the least important imperative, which contains the global weight of 0.0401.

## 5. Discussions

The global ranking (Fig 7) suggests that the "Availability of government support schemes to promote sustainability (I16)", "Developing strategic supply chain interventions to ameliorate the impact of disruptive events (I1)", and "Digitalizing the SC (I4)" are the topmost important imperatives. From an economic standpoint, the emphasis on the "Availability of government support schemes to promote sustainability (I16)" as illustrated in Fig 4, highlights their critical role in stabilizing the supply chain during crises. Government interventions, through strategic policies and financial aid, are instrumental in mitigating the adverse impacts of global disruptions on supply chains. This finding is supported by Das et al. [70], Chatterjee & Chaudhuri [71], and Karmaker et al. [41], who note the essential nature of government strategies in ensuring the continuity of supply and maintaining public trust. The literature suggests that beyond immediate crisis management, these policies are vital for long-term economic stability and supply chain resilience, as echoed by Lee [72] and further corroborated by Ivanov & Dolgui [35], who highlighted the strategic significance of supply chains as policy tools for national security and resilience.

"Digitizing the SC (I4)", identified as a crucial imperative, underscores the transformative power of digital technologies in enhancing efficiency and adaptability. This aspect is particularly relevant in the context of the Bangladeshi fashion retail industry, where digital solutions like blockchain technology offer unprecedented opportunities for transparency, efficiency, and stakeholder trust [55, 54]. The integration of digital technologies facilitates agile decision-making and robust capacity management, which is essential for sustaining supply chain operations during and beyond disruptive events [57, 73]. Furthermore, the advent of AI and robotics in logistics and inventory management heralds a new era of supply chain optimization, contributing to environmental sustainability and operational resilience [41, 74].

The imperative "Developing strategic SC interventions to ameliorate the impact of disruptive events (I1)" underscores the need for a proactive and strategic approach to supply chain management. This strategy is vital for addressing the social implications of disruptions, such as job losses and community destabilization, emphasizing the importance of maintaining diverse supply sources and effective communication with government bodies [75, 76]. The study also highlights the growing significance of "Increasing the application of circular manufacturing(I13)" imperative in promoting environmental and social sustainability within supply chains. By focusing on resource efficiency and circularity, these practices contribute to a more sustainable and resilient supply chain ecosystem, addressing key concerns related to consumer rights and environmental stewardship [77, 78]. This approach not only improves environmental quality but also addresses consumer rights and recycling issues [79, 80]. Enhancing stakeholder collaboration is pivotal in strengthening SC security and operational capabilities. Collaborative approaches in planning and execution can lead to better visibility, traceability, and data exchange [81, 82].

Elevating consumer awareness about eco-friendly products is vital for encouraging shifts towards more sustainable consumer practices [83]. Fig 6 reveals that "Enhancing customer understanding of sustainable products (I3)" ranks as the foremost environmental priority. Meanwhile, "Integrating robotics in logistics and inventory management (I6)" stands as the second most crucial environmental imperative for supply chain sustainability (SCS) within the context of the Bangladeshi fashion retail sector. The adoption of robotics and artificial intelligence (AI) in logistics and manufacturing processes plays a significant role in boosting supply chain efficiency and resilience, thereby aiding environmental sustainability efforts [74, 41].

Enhancing the resilience of transportation and logistics is critical, especially against the backdrop of environmental challenges brought on by disruptions such as pandemics and geopolitical unrest. The application of AI in manufacturing processes enhances collaboration among stakeholders and enables the swift identification of disruptions, facilitating quick adjustments to alternative suppliers [41]. Moreover, AI improves logistics and inventory management and aids in demand forecasting, leading to reduced costs, more efficient resource utilization, and heightened productivity. Consequently, our research identifies "Applying Artificial Intelligence (AI) in manufacturing systems (I15)" as a key environmental imperative.

This study’s insights stand out when compared to other contemporary research in the domain of SCS, particularly given the unique challenges faced by the Bangladeshi fashion retail industry amid pandemics and geopolitical strife. Gupta et al. [81] highlighted the role of structured management and decision-making support in navigating barriers to SCS innovation, though their analysis remains broad and less specific to the fashion retail industry’s challenges. Karmaker et al. [41] focused on immediate drivers during the COVID-19 pandemic, such as policy formulation, health protocols, and governmental financial backing, without delving into the long-term strategies necessary for supply chain resilience. Likewise, Nasir et al. [57] offered policy suggestions for sustaining small and medium-sized enterprises during the pandemic, which might not fully resonate with the particular needs of the fashion retail sector.

Our study, however, illuminates three critical imperatives—customized government support schemes for sustainability, strategic supply chain interventions for enduring resilience, and in-depth insight into consumer awareness of sustainable products. These elements offer detailed, industry-specific guidance for decision-makers within the Bangladeshi fashion retail sector, laying out a holistic framework to tackle the distinct challenges presented by pandemics and geopolitical tensions. This approach not only focuses on immediate financial and policy assistance but also highlights the significance of strategic planning, consumer behavior analysis, and the pursuit of long-term supply chain sustainability, presenting a unique and valuable viewpoint within the sphere of SCS.

### 5.1 Theoretical implications

The examination of disruptions within supply chains, although a subject of scientific scrutiny for many years, has gained unprecedented attention in light of recent global events such as COVID-19 and the Russia-Ukraine conflict. This heightened focus underscores an acute necessity for in-depth research into distinctive and novel disruptive phenomena alongside the formulation of sustainability models adept at navigating these challenges. The importance of SCS research amidst such disruptions is accentuated by a burgeoning demand for quantitative decision-making models. These models are envisioned to synergize with new digital technologies, substantial government support, and a plethora of strategies aimed at mitigating disruption impacts. This investigation stands as one of the initial endeavors to meticulously dissect SCS imperatives through a tripartite lens—economic, social, and environmental—thereby offering a holistic view of the multifaceted challenges and solutions within the realm of supply chain management during tumultuous periods. By delving into the critical requirements for SCS amid significant disruptions, such as the COVID-19 pandemic and the geopolitical strife stemming from Russia’s invasion of Ukraine, this research not only validates the imperative of scrutinizing supply chain disruptions but also pioneers a structured framework for understanding and mitigating these challenges.

Utilizing a novel MCDM methodological framework, such as the Bayesian BWM, this study delineates the essential criteria for adeptly navigating through disruptive events. This methodology signifies a unique scholarly contribution, delineating the pivotal components necessary for the effective management and fortification of SCS in the inherently unstable fashion retail industry landscape. Moreover, by offering an exhaustive inventory of crucial imperatives, evaluated from diverse perspectives, this study emboldens scholars to undertake thorough examinations of each imperative, propelling a more nuanced comprehension of the critical SCS strategies essential for maintaining operational efficacy and diminishing adverse impacts amid disruptions.

Furthermore, this study pioneers in establishing a nexus between SCS, its imperatives, and the varying sustainability perspectives in the context of recent disruptions, charting a novel course in the exploration of supply chain resilience and sustainability. This endeavor not only illuminates the path for future research but also equips practitioners and policymakers with the insights necessary for crafting supply chains that are both resilient and sustainable, even in the face of unrelenting global challenges.

### 5.2 Practical implications

The practical implications of SCS span across business operations, societal welfare, and environmental stewardship. The urgency to balance these dimensions becomes particularly critical in the face of disruptive events such as pandemics and geopolitical conflicts, which test the resilience and competitiveness of industries, notably the fashion retail sector. This study’s insights offer actionable strategies for navigating such uncertainties, emphasizing the integration of sustainability into core business practices to ensure not only survival but also long-term success.

In addressing the challenges posed by events like COVID-19 and the Russia-Ukraine conflict, our findings underscore the necessity of a robust SCS framework. For industry leaders in Bangladesh’s fashion retail sector, the study outlines a blueprint for maintaining competitiveness through resilience, focusing on pivotal areas such as government support for sustainability initiatives and the digital transformation of supply chains. The role of government in providing tailored support and facilitating trade activities is crucial, highlighting the need for policies that foster a conducive environment for sustainable practices and innovation. Digitalization emerges as a cornerstone for enhancing SCS, with technologies like blockchain and artificial intelligence streamlining operations and improving transparency, efficiency, and product quality. These technological advancements not only support operational excellence but also contribute to environmental sustainability by optimizing resource use and reducing waste.

### 5.3 Implications for SDGs

This section elaborates on how SCS serves as a critical lever for addressing both immediate and long-term global sustainability challenges. The integration of advanced technologies and sustainable practices within supply chains has the potential to catalyze significant progress across multiple SDGs by fostering economic growth, improving health and safety standards, ensuring food security, and promoting sustainable consumption and production patterns.

The deployment of big data analytics and artificial intelligence (AI) within supply chains represents a transformative shift towards more efficient, responsive, and intelligent supply chain management. By optimizing logistics and production processes, these technologies can significantly reduce waste and increase operational efficiency, leading to cost savings and improved productivity. This contributes directly to SDG 8 (Decent Work and Economic Growth) by creating more resilient and prosperous economic conditions, and indirectly supports SDG 1 (No Poverty) by generating economic opportunities and potentially lowering the cost of goods for consumers. Furthermore, the emphasis on digitalization and circular manufacturing practices can spur innovation and infrastructure development (SDG 9), providing a foundation for sustainable industrialization that minimizes environmental impact while fostering economic growth.

The push towards sustainable consumption and production (SDG 12) is significantly bolstered by the adoption of SCS practices. Encouraging the use of sustainable, eco-friendly products through supply chain transparency and consumer engagement can drive a shift in market demand, incentivizing companies to adopt greener practices. Government initiatives that support sustainability in supply chains—through incentives, regulations, or direct support—play a crucial role in this transition. Through the lens of SCS, the study also underscores the potential for supply chains to contribute to the development of inclusive, resilient communities (SDG 11). By promoting practices that are not only environmentally sustainable but also socially inclusive, supply chains can help ensure that economic benefits are widely distributed and that all members of society have access to the opportunities created by sustainable development.

## 6. Conclusion

This study delves into the complexities of SCS in the face of global disruptions such as the COVID-19 pandemic and the Russia-Ukraine conflict. These unprecedented events have underscored the fragility of global supply chains, presenting a unique challenge for policymakers worldwide. Addressing this urgent issue requires identifying, assessing, and prioritizing imperatives that support the adoption and implementation of SSC practices. Through a comprehensive review of research publications and expert questionnaires, this study has identified 18 key sustainability imperatives impacting supply chains. Subsequent Pareto Analysis refined this list to twelve critical imperatives for in-depth exploration.

Utilizing the Bayesian-BWM, the study highlights "Availability of government support schemes to promote sustainability (I16)" as the most critical imperative from an economic perspective, underlined by its high confidence level. This is followed closely by imperatives like "Digitizing the SC (I4)", "Deploying blockchain technology in SC management (I12)", and "Increasing the application of big data analytics (BDA) (I2)". On the social front, "Developing strategic SC interventions to ameliorate the impact of disruptive events (I1)" emerges as the top priority, with other significant imperatives including "Ensuring compliance with health and safety legislations across the SC (I10)", "Increasing the application of circular manufacturing (I13)", and "Improving collaboration among SC stakeholders (I5)". From an environmental perspective, the study underscores "Understanding customer awareness of sustainable products (F3)" as paramount, followed by the integration of robotics in logistics (I6), the use of AI in manufacturing systems (I15), and enhancing the resilience of transportation and logistics systems (I17).

This research pioneers the linking of SCS with the effects of COVID-19 and geopolitical disruptions, offering a novel framework based on Pareto analysis and Bayesian-BWM for analyzing critical SCS imperatives. This framework is instrumental for decision-makers, aiding in strategizing to enhance SC resilience amid current disruptions. The study also highlights the severe impact of recent disruptions on SC stability and the vital imperatives necessary to navigate these challenges effectively.

In this study, the prioritization of sustainability imperatives was based on expert judgment. This approach is widely used in sustainability research, especially when managers have to deal with complex and uncertain issues. While expert input provides valuable insights, we recognize that it may not always represent an absolute truth about what is “most important” for improving sustainability. Instead, it offers a perspective grounded on practical experience and theoretical knowledge. Expert decision-making is often used when data is scarce or difficult to gather and when nuanced understanding is needed for improved decision-making [2, 23]. Previously published works also show that expert opinions can reliably guide while making decisions, particularly in areas related to ‘sustainability research’, where data is scarce, and decision-makers often need to rely on experience, contextual knowledge, and technical expertise [2, 23, 59]. Thus, the contribution of this study is to provide insights into how experts prioritize sustainability imperatives, and the obtained results reflect expert perspectives, offering a credible foundation for decision-making, even though these are not definitive claims about what is universally "most important" for achieving sustainability.

This study also acknowledges certain limitations in data collection, focusing only on twelve imperatives and relying on expert recommendations that may introduce bias. Future research could expand the scope to more imperatives and utilize Structural Equation Modeling (SEM) for a more comprehensive analysis. Additionally, exploring the interplay of these imperatives using Grey-based Total Interpretive Structural Modelling (TISM) could offer deeper insights. While the study primarily targets the garment manufacturing SCs in developing countries, its implications are far-reaching, impacting various global industries affected by the Russia-Ukraine conflict. The methodologies and findings of this study have the potential to significantly influence the broader discourse on SCS in the context of global disruptions.

## Supporting information

S1 File Available in the submitted "S1 File". (DOCX)
