# Supplementary material for: Modeling the supply chain sustainability imperatives in the fashion retail industry: Implications for sustainable development
Source: PLoS One. 2024 Dec 31;19(12):e0312671. doi: 10.1371/journal.pone.0312671 (PMC11687714; doi:10.1371/journal.pone.0312671)
Supplement: S1 File — (DOCX) [file pone.0312671.s001.docx]

## **Supporting information file (S1 file)**

## **Appendix A**

**Primary Questionnaire**

**The questionnaire for the survey is presented as follows:**

- What role do you represent in the fashion retail industry?
- Mention the years of experience you have in the fashion retail industry.
- Please select the imperatives according to their relevance. If you think any imperatives are irrelevant, you can remove the imperative from this list and add new imperatives at the end of this existing list.

| **No.** | **Imperatives of SCS** | **Is it relevant?**  **(Yes/No)** |
| --- | --- | --- |
| 01 | Developing strategic SC interventions to ameliorate the impact of disruptive events |  |
| 02 | Increasing the application of big data analytics (BDA) |  |
| 03 | Understanding the customer awareness of sustainable products |  |
| 04 | Digitizing the SC |  |
| 05 | Improving the collaboration among SC stakeholders |  |
| 06 | Exploding the use of robotics in logistics and inventory system |  |
| 07 | Development of intertwined supply network (ISN) |  |
| 08 | Increasing the use of the Internet of Things (IoT) in the SC activities |  |
| 09 | Integrating cloud manufacturing technologies |  |
| 10 | Prepare for the rebound |  |
| 11 | Ensuring compliance with the health and safety legislations across the SC |  |
| 12 | Increasing the localized production capability to buffer disruptions. |  |
| 13 | Deploying blockchain technology in SC management |  |
| 14 | Increasing the application of circular manufacturing |  |
| 15 | Strategic planning for sustainability |  |
| 16 | Improving the public-private relationships |  |
| **Add new relevant imperatives (if any)** | | |
|  |  | |
|  |  | |
|  |  | |

**Questionnaire for Pareto analysis:**

**The questionnaire for the Pareto analysis is presented as follows:**

Ratings are provided on a priority basis. Participants were asked to rate each imperative on a scale of 1 to 5, where 1 represented the least important imperative, and 5 represented the most important imperative.

| **Imperatives of SCS** | **Code** | **Give a rating (1-5) based on your priority.** | | | | |
| --- | --- | --- | --- | --- | --- | --- |
|  |  | Not Important | Less Important | Important | More Important | Most Important |
|  |  | **1** | **2** | **3** | **4** | **5** |
| Developing strategic SC interventions to ameliorate the impact of disruptive events | I1 |  |  |  |  |  |
| Increasing the application of big data analytics (BDA) | I2 |  |  |  |  |  |
| Understanding the customer awareness of sustainable products | I3 |  |  |  |  |  |
| Digitizing the SC | I4 |  |  |  |  |  |
| Improving the collaboration among SC stakeholders | I5 |  |  |  |  |  |
| Exploding the use of robotics in logistics and inventory system | I6 |  |  |  |  |  |
| Development of intertwined supply network (ISN) | I7 |  |  |  |  |  |
| Increasing the use of the Internet of Things (IoT) in the SC activities | I8 |  |  |  |  |  |
| Integrating cloud manufacturing technologies | I9 |  |  |  |  |  |
| Ensuring compliance with the health and safety legislations across the SC | I10 |  |  |  |  |  |
| Increasing the localized production capability to buffer disruptions | I11 |  |  |  |  |  |
| Deploying blockchain technology in SC management | I12 |  |  |  |  |  |
| Increasing the application of circular manufacturing | I13 |  |  |  |  |  |
| Improving the public-private relationships | I14 |  |  |  |  |  |
| Using Artificial Intelligence (AI) in manufacturing system | I15 |  |  |  |  |  |
| Availability of government support schemes to promote sustainability | I16 |  |  |  |  |  |
| Enhancing the resilience of transportation and logistics system | I17 |  |  |  |  |  |
| Enabling a scattered supplier management system | I18 |  |  |  |  |  |

## **Appendix B**

Determination of best and worst imperatives of the SCS by the experts from an economic perspective.

| **Code** | **Imperatives of (SSC)** | **Determined as “Best” by the expert No.** | **Determined as “Worst” by expert No.** |
| --- | --- | --- | --- |
| I4 | Digitizing the SC | 1,5 | 4 |
| I2 | Increasing the application of big data analytics (BDA) |  | 5,6,7,8 |
| I16 | Availability of government support schemes to promote sustainability | 2,3,7,8 |  |
| I12 | Deploying blockchain technology in SC management | 4 | 1,2,3 |

Determination of best and worst imperatives of the SCS by the experts from a social perspective.

| **Code** | **Imperatives of (SSC)** | **Determined as “Best” by the expert No.** | **Determined as “Worst” by expert No.** |
| --- | --- | --- | --- |
| I1 | Developing strategic SC interventions to ameliorate the impact of disruptive events | 1,7,8 |  |
| I5 | Improving the collaboration among SC stakeholders | 4 | 2,3,6,8 |
| I10 | Ensuring compliance with the health and safety legislations across the SC | 2,3 | 5 |
| I13 | Increasing the application of circular manufacturing | 5,6 | 1,4,7 |

Determination of best and worst imperatives of the SCS by the experts from an environmental perspective.

| **Code** | **Imperatives of (SSC)** | **Determined as “Best” by the expert No.** | **Determined as “Worst” by expert No.** |
| --- | --- | --- | --- |
| I3 | Understanding the customer awareness of sustainable products | 2,5,6 | 4 |
| I15 | Using Artificial Intelligence (AI) in manufacturing system | 1,3 | 2 |
| I6 | Exploding the use of robotics in logistics and inventory system | 4,7 | 5,8 |
| I17 | Enhancing the resilience of transportation and logistics system | 8 | 1,3,6,7, |

Determination of best and worst factors of the SCS by evaluators for main criteria.

| **Criteria** | **Determined as “Best” by the expert No.** | **Determined as “Worst” by expert No.** |
| --- | --- | --- |
| Economic | 2,5,6,8 | 4 |
| Social | 1,3,4 | 2,5,7 |
| Environmental | 7 | 1,3,6,8 |

Determining the preference of the best criteria over all others from the economic perspective.

| Evaluator | Best | I4 | I2 | I16 | I12 |
| --- | --- | --- | --- | --- | --- |
| Evaluator 1 | I4 | 1 | 9 | 2 | 5 |
| Evaluator 2 | I16 | 4 | 8 | 1 | 6 |
| Evaluator 3 | I16 | 5 | 7 | 1 | 6 |
| Evaluator 4 | I12 | 3 | 8 | 2 | 1 |
| Evaluator 5 | I4 | 1 | 8 | 2 | 6 |
| Evaluator 6 | F11 | 3 | 9 | 5 | 7 |
| Evaluator 7 | I16 | 1 | 8 | 1 | 6 |
| Evaluator 8 | I16 | 4 | 6 | 1 | 7 |

Determining the preference of the best criteria over all others from the social perspective.

| Evaluator | Best | I1 | I5 | I10 | I13 |
| --- | --- | --- | --- | --- | --- |
| Evaluator 1 | I1 | 1 | 5 | 4 | 6 |
| Evaluator 2 | I10 | 3 | 5 | 1 | 7 |
| Evaluator 3 | I10 | 3 | 5 | 1 | 8 |
| Evaluator 4 | I5 | 2 | 1 | 4 | 7 |
| Evaluator 5 | I13 | 2 | 7 | 3 | 1 |
| Evaluator 6 | I13 | 2 | 8 | 3 | 1 |
| Evaluator 7 | I1 | 1 | 5 | 4 | 6 |
| Evaluator 8 | I1 | 1 | 6 | 4 | 8 |

Determining the preference of the best criteria over all others from the environmental perspective.

| Evaluator | Best | I3 | I15 | I6 | I17 |
| --- | --- | --- | --- | --- | --- |
| Evaluator 1 | I15 | 2 | 1 | 4 | 7 |
| Evaluator 2 | I3 | 1 | 4 | 5 | 8 |
| Evaluator 3 | I15 | 2 | 1 | 5 | 7 |
| Evaluator 4 | I6 | 2 | 5 | 1 | 7 |
| Evaluator 5 | I3 | 1 | 5 | 4 | 8 |
| Evaluator 6 | I3 | 1 | 4 | 5 | 6 |
| Evaluator 7 | I6 | 2 | 4 | 1 | 7 |
| Evaluator 8 | I17 | 3 | 5 | 6 | 1 |

Determining the preference of the worst criteria over all others from the economic perspective.

| Evaluator | Evaluator 1 | Evaluator 2 | Evaluator 3 | Evaluator 4 | Evaluator 5 | Evaluator 6 | Evaluator 7 | Evaluator 8 |
| --- | --- | --- | --- | --- | --- | --- | --- | --- |
| Worst  criteria | I12 | I12 | I12 | I4 | I2 | I2 | I2 | I2 |
| I4 | 5 | 6 | 5 | 1 | 5 | 4 | 4 | 6 |
| I2 | 2 | 3 | 3 | 2 | 1 | 1 | 1 | 1 |
| I16 | 8 | 8 | 9 | 9 | 8 | 7 | 9 | 7 |
| I12 | 1 | 1 | 1 | 3 | 4 | 6 | 4 | 5 |

Determining the preference of the worst criteria over all others from the social perspective.

| Evaluator | Evaluator 1 | Evaluator 2 | Evaluator 3 | Evaluator 4 | Evaluator 5 | Evaluator 6 | Evaluator 7 | Evaluator 8 |
| --- | --- | --- | --- | --- | --- | --- | --- | --- |
| Worst  criteria | I13 | I5 | I5 | I13 | I10 | I5 | I13 | I5 |
| I1 | 8 | 9 | 8 | 9 | 8 | 9 | 8 | 7 |
| I5 | 3 | 1 | 1 | 2 | 2 | 1 | 3 | 1 |
| I10 | 5 | 5 | 4 | 4 | 1 | 5 | 5 | 6 |
| I13 | 1 | 4 | 6 | 1 | 5 | 6 | 1 | 5 |

Determining the preference of the worst criteria over all others from the environmental perspective.

| Evaluator | Evaluator 1 | Evaluator 2 | Evaluator 3 | Evaluator 4 | Evaluator 5 | Evaluator 6 | Evaluator 7 | Evaluator 8 |
| --- | --- | --- | --- | --- | --- | --- | --- | --- |
| Worst  criteria | I17 | I15 | I17 | I3 | I6 | I17 | I17 | I6 |
| I3 | 5 | 4 | 4 | 1 | 4 | 5 | 4 | 5 |
| I15 | 4 | 1 | 5 | 5 | 5 | 6 | 5 | 4 |
| I6 | 6 | 5 | 7 | 6 | 1 | 5 | 7 | 1 |
| I17 | 1 | 9 | 1 | 7 | 8 | 1 | 1 | 7 |

Determining the preference of the best criteria over all others from the main criteria.

| Evaluator | Best | Economic | Social | Environmental |
| --- | --- | --- | --- | --- |
| Evaluator 1 | Social | 3 | 1 | 8 |
| Evaluator 2 | Economic | 1 | 4 | 8 |
| Evaluator 3 | Social | 2 | 1 | 9 |
| Evaluator 4 | Social | 3 | 1 | 7 |
| Evaluator 5 | Economic | 1 | 6 | 7 |
| Evaluator 6 | Economic | 1 | 5 | 9 |
| Evaluator 7 | Environmental | 2 | 7 | 1 |
| Evaluator 8 | Economic | 1 | 4 | 9 |

Determining the preference of the worst criteria over all others for the main criteria.

| Evaluator | Evaluator 1 | Evaluator 2 | Evaluator 3 | Evaluator 4 | Evaluator 5 | Evaluator 6 | Evaluator 7 | Evaluator 8 |
| --- | --- | --- | --- | --- | --- | --- | --- | --- |
| Worst  criteria | Environmental | Social | Environmental | Economic | Social | Environmental | Social | Environmental |
| Economic | 3 | 2 | 2 | 1 | 3 | 3 | 2 | 2 |
| Social | 6 | 1 | 7 | 5 | 1 | 7 | 1 | 5 |
| Environmental | 1 | 8 | 1 | 9 | 8 | 1 | 9 | 1 |

## **Appendix C**

The profile of the experts is as follows:

| **Number** | **Designation** | **Experience** | **Job Responsibility** |
| --- | --- | --- | --- |
| Expert 1 | Assistant Professor | More than 5 years | Teaching |
| Expert 2 | Assistant General Manager | More than 15 years | Merchandising & Marketing |
| Expert 3 | Deputy General Manager | More than 15 years | Production |
| Expert 4 | Assistant Manager | More than 10 years | Operations |
| Expert 5 | Manager | More than 15 years | Technical Operations |
| Expert 6 | Assistant Manager | More than 5 years | Industrial Engineering |
| Expert 7 | Sr. Executive | More than 5 years | Environmental Health Safety & Sustainability |
| Expert 8 | Associate Professor | More than 8 years | Teaching |
| Expert 9 | General Manager | More than 20 years | Supply Chain |
| Expert 10 | Assistant General Manager | More than 15 years | Production |
| Expert 11 | Deputy General Manager | More than 18 years | Environmental Health Safety & Sustainability |
| Expert 12 | Manager | More than 15 years | Operations |
| Expert 13 | Senior Manager | More than 15 years | Operations |
| Expert 14 | Lecturer | More than 4 years | Teaching |
| Expert 15 | Assistant Manager | More than 10 years | Environmental Health Safety & Sustainability |
| Expert 16 | Manager | More than 15 years | Industrial Engineering |
| Expert 17 | General Manager | More than 20 years | Merchandising & Marketing. |
| Expert 18 | Deputy General Manager | More than18 years | Supply Chain |
| Expert 19 | Assistant Manager | More than 10 years | Production |
| Expert 20 | Manager | More than 15 years | Quality Assurance |
| Expert 21 | Senior Manager | More than 18 years | Logistics Management |
| Expert 22 | Deputy Manager | More than 8 years | Maintenance |
| Expert 23 | General Manager | More than 20 years | Supply Chain |
| Expert 24 | Deputy General Manager | More than 18 years | Compliance, HR & Admin |
